# Supplementary material for: The Construction and Exploration of a Comprehensive MicroRNA Centered Regulatory Network in Foxtail Millet (Setaria italica L.)
Source: Front Plant Sci. 2022 May 6;13:848474. doi: 10.3389/fpls.2022.848474 (PMC9121102; doi:10.3389/fpls.2022.848474)
Supplement: Supplementary file 4 [file Data_Sheet_4.zip › Sit-miRN3020_Seita.2G170300.1_156_TPlot.pdf]

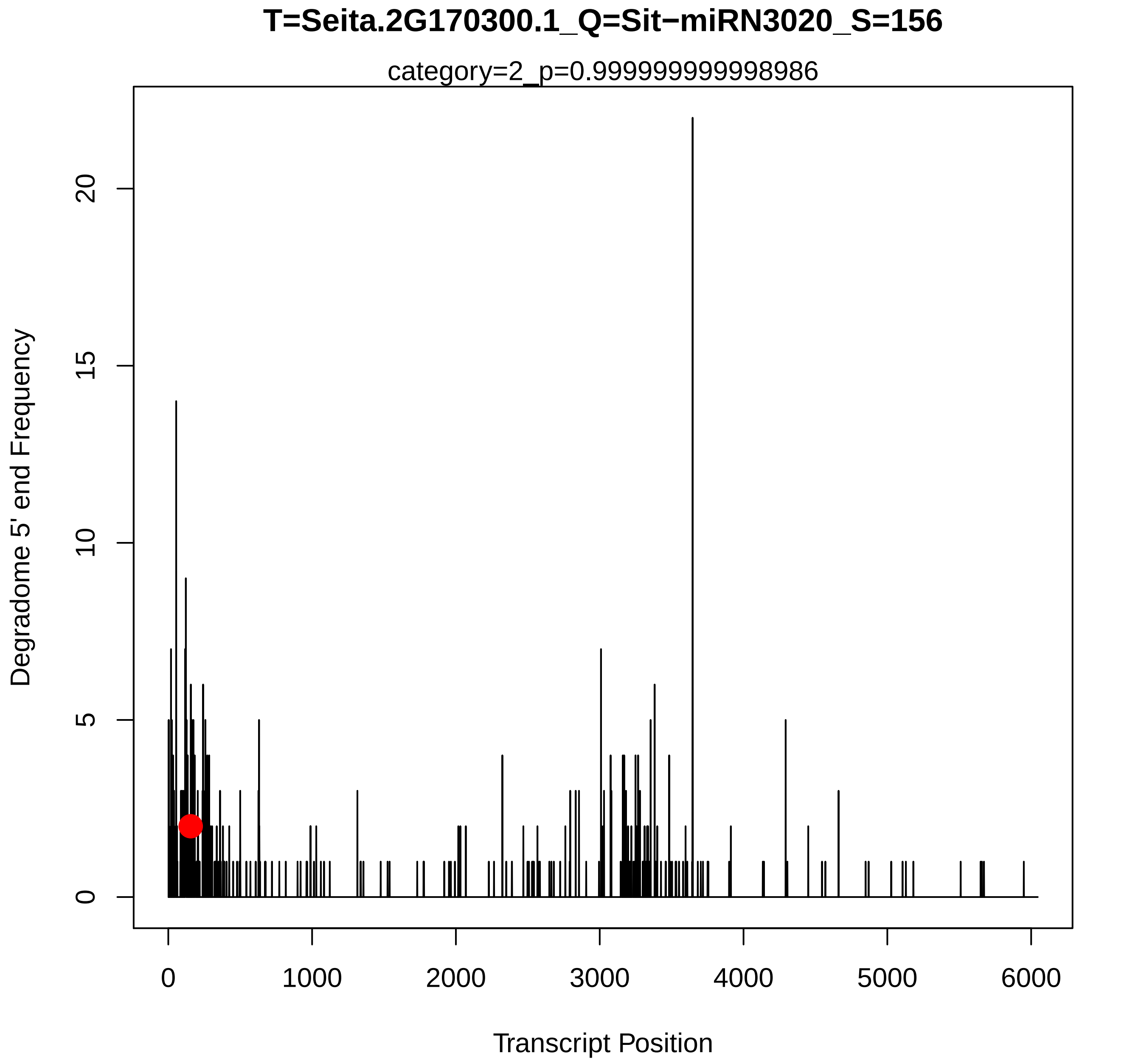
T=Seita.2G170300.1_Q=Sit−miRN3020_S=156
category=2_p=0.999999999998986
Degradome 5' end Frequency
0
5
10
15
20
0
1000
2000
3000
4000
5000
6000
Transcript Position
